# Hyperplasie macronodulaire bilatérale des surrénales: cause rare du syndrome de Cushing (à propos d’un cas)

**DOI:** 10.11604/pamj.2021.39.98.26041

**Published:** 2021-06-02

**Authors:** Yassine Er-rahali, Amal Moumen, Souad Elmoussaoui, Ahmed Anass Guerboub, Ghizlaine Belmejdoub

**Affiliations:** 1 Service d’Endocrinologie - Diabétologie, Hôpital Militaire d’instruction Mohammed V, Faculté de Médecine et de Pharmacie, Université Mohammed V, Souissi, Rabat, Maroc

**Keywords:** Macronodular adrenal hyperplasia, Cushing's syndrome, adrenalectomy, case report, Hyperplasie macronodulaire des surrénales, syndrome de Cushing, surrénalectomie, à propos d’un cas

## Abstract

L´hyperplasie macronodulaire bilatérale des surrénales (HMBS) est une cause rare de syndrome de Cushing d´origine surrénalienne, représentant moins de 1% des cas. Nous rapportons le cas d´un patient âgé de 48 ans, diabétique et hypertendu. Présentant un syndrome de Cushing clinique. Le bilan étiologique a permis de retenir le diagnostic d´hypercorticisme «Adrenocorticotropic hormone» (ACTH) indépendant en rapport avec une HMBS. Le choix thérapeutique était en faveur d´une surrénalectomie unilatérale gauche orientée par la scintigraphie au noriodocholestérol, avec une bonne évolution. Cependant, vu le risque de récidive et de complications cardiovasculaires, une surveillance au long cours a été programmée.

## Abstract (en)

Bilateral macronodular adrenal hyperplasia (BMAH) is a rare cause of adrenal Cushing's syndrome, accounting for less than 1% of cases. We here report the case of a 48-year-old patient with diabetes and hypertension presenting with clinical signs of Cushing's syndrome. Etiological assessment enabled clinicians to retain the diagnosis of Adrenocorticotropic hormone (ACTH)-independent hypercortisolism associated with BMAH. Unilateral left adrenalectomy was performed based on noriodocholesterol scintigraphy results, with good outcome. However, given the risk of recurrence and cardiovascular complications, long-term monitoring was scheduled.

## Introduction

Le syndrome de Cushing correspond à une sécrétion inappropriée est chronique de glucocorticoïde, il est dû à une sécrétion autonome d´origine surrénalienne dans 15% des cas il est dit alors ACTH indépendant. Ses principales étiologies sont l´adénome surrénalien et le corticosurrénalome malin, l´hyperplasie macronodulaire bilatérale des surrénales en est une cause rare représentant moins de 1% des cas [1].

## Patient et observation

Nous rapportant le cas d´un patient âgé de 48 ans, diabétique de type 2 depuis 8 ans, mal équilibré (HbA1c à 9.6%) sous insuline glargine+ metformine + sulfamide hypoglycémiant, hypertendu depuis dix ans sous trithérapie (Ramipril + HCT+ Amlodipine). Il présentait depuis 6 mois une symptomatologie évocatrice d´un syndrome de Cushing fait d´une obésité facio-tronculaire, une érythrose faciale, comblement des creux sus claviculaire et des vergetures pourpres niveau de l´abdomen. Le bilan endocrinien a objectivé une rupture de cycle nycthéméral du cortisol avec un test de freinage-minute négatif (cortisolémie de 8h après prise de 1mg de Déxaméthasone à minuit était à 135ng/ml). Un dosage de cortisol libre urinaire est revenu positif avec une valeur supérieure à 3 fois la limite supérieure de la normale à deux reprises ce qui a permis de poser le diagnostic d´hypercorticisme.

**Figure 1:**
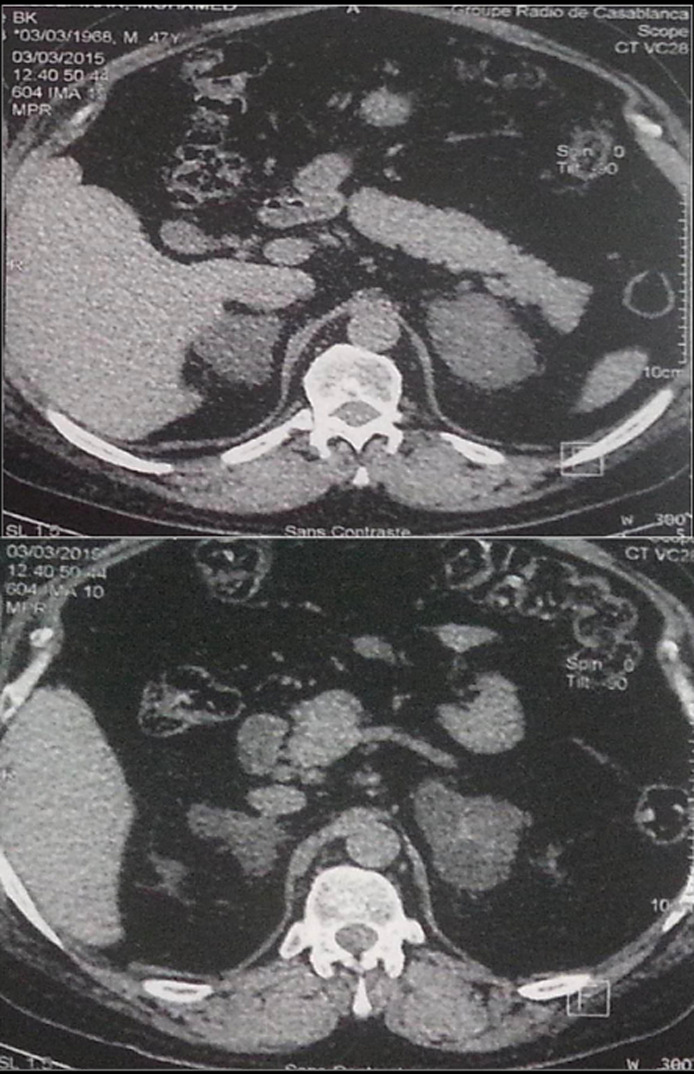
TDM surrénalienne du patient montrant l’hyperplasie macronodulaire bilatérale des surrénales

**Figure 2:**
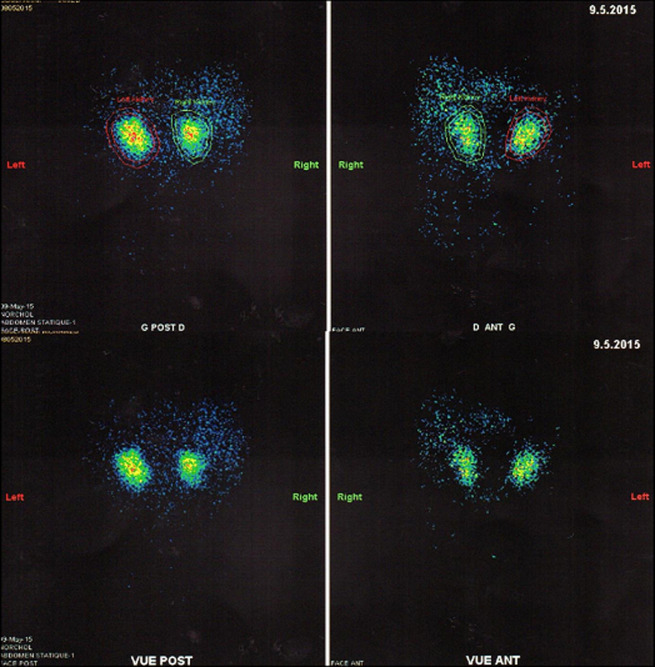
scintigraphie surrénalienne à l’iodocholestérol, montant une hyperfixation des deux surrénales en faveur d’une hyperplasie surrénalienne bilatérale, la surrénale gauche parait plus active que la droite (58% à gauche vs 42% à droite)

Devant un taux d´ACTH plasmatique effondré (ACTH <0,2) l´origine périphérique de l´hypercorticisme a été retenue. Le bilan morphologique fait d´une TDM surrénalienne a mis en évidence des surrénales très augmentée de taille par la présence de multiples macro-nodules (Figure 1) en faveur d´une hyperplasie macronodulaire bilatérale des surrénales. La scintigraphie au 19-noriodocholesterol a montré un aspect en faveur d´une hyperplasie bilatérale des surrénales avec une fixation légèrement plus intense du côté gauche (58% à gauche vs 42% à droite) (Figure 2). L´IRM hypothalamo-hypophysaire était sans particularité.

Le patient a bénéficié d´une surrénalectomie unilatérale gauche dont l´étude anatomopathologique a confirmé le diagnostic en concluant à une hyperplasie corticosurrénalienne macronodulaire, par ailleurs absence d´expression d´anticorps anti-ACTH. L´évolution a été favorable avec amélioration du diabète (équilibré sous metformine seule) et de l´HTA actuellement équilibrée sous Ramipril 5mg/j. Sur le plan hormonal, le cortisol libre urinaire dosé à 3 puis à 6 mois du post-op était normal. Le patient est suivi régulièrement en consultation et garde jusqu´à présent des valeurs du cortisol libre urinaire dans la limite de la normale de même qu´une réponse positive au test de freinage-minute. Un suivi au long cours est prévue afin de décelé une éventuelle rechute.

## Discussion

L´HMBS correspond à des masses multi-nodulaires surrénaliennes bénignes, bilatérales, volumineuses (Figure 1) et responsables d´un SC patent ou plus fréquemment d´un hypercorticisme à minima dit «infra clinique». Dans ce cas, l´HMBS est volontiers découverte fortuitement [2]. Hormis des rares cas qui se développent à un âge précoce, souvent associés à des anomalies génétiques notamment le syndrome de McCune Albright, la majorité des cas sont sporadiques et se développent entre la 5^e^ et la 6^e^ décennie, ce qui est le cas de notre patient, avec répartition égale entre les deux sexes [3]. Le mécanisme physiopathologique des HMBS était longuement méconnu. Mais depuis les années 1990, on sait que dans de nombreux cas d´HMBS, la sécrétion cortisonique des cellules hyperplasiques fait l´objet d´une régulation aberrante médiée par l´expression ou l'altération de l'activité des récepteurs eutociques comme ceux de la vasopressine (récepteur V1-vasopressine), hormone lutéinisante/gonadotrophine chorionique humaine (LH/hCGR), la sérotonine (récepteur 5-HT4) et le récepteur de la leptine [3].

En 2003, Lefebvre *et al*. ont objectivé la présence et la sécrétion d´ACTH au niveau de cellules corticosurrénaliennes hyperplasiques, associée à une ACTH plasmatiques effondrée [4]. Cette ACTH locale va agir selon un mode autocrine et paracrine soit directement sur ses propres récepteurs ou indirectement en amplifiant l'action des ligands des récepteurs aberrants [5]. Chez notre patient la recherche des récepteurs illégitimes n'a pas été réalisée, mais l'étude immune histochimique de la pièce opératoire n'a pas révélée la présence d´ACTH locale. Enfin l´HMBS a probablement une origine génétique comme le suggère une étude publiée en 2013 par Assié *et al*. qui a objectivé la présence de mutations du gène ARMC5 dans 50% des cas d´HMBS étudiés [6].

Le diagnostic d´hyperplasie macronodulaire repose sur le bilan hormonal et morphologique d´un patient présentant un syndrome de Cushing. Le bilan endocrinien permet de mettre en évidence un syndrome de Cushing d´origine surrénalienne et autonome. Il permet d'objectiver un hypercorticisme avec une élévation du cortisol libre dans les urines et une perte du rythme circadien. Le taux plasmatique d´ACTH est indétectable en condition basale indiquant que ces désordres sont d´origine périphérique [7]. Sur la TDM (examen tomodensitométrique) on trouve des surrénales très augmentées de taille et multi nodulaires, la densité spontanée est basse évoquant le caractère bénin de l'hyperplasie. En imagerie par résonance magnétique (IRM) les surrénales hypertrophiées donnent un signal moins intense que le foie en T1 et un signal hyper-intense en T2. La scintigraphie à l´iodométhylnorcholestérol montre une fixation bilatérale [4]. L'IRM hypothalamo hypophysaire ne retrouve aucune anomalie.

La surrénalectomie bilatérale était pendant longtemps un traitement de choix, telle procédure expose le patient à une insuffisance surrénalienne impliquant une opothérapie substitutive à vie avec une altération importante de la qualité de vie et peut même engager le pronostic vital lors d'une décompensation aigue [8]. Récemment la surrénalectomie unilatérale a pris le devant comme traitement de première ligne. Plusieurs études récentes en rapporter l´efficacité de cette approche dans le traitement de l´HMBS [9]. En effet la surrénalectomie unilatérale à un taux de succès de 85% avec un risque d´insuffisance surrénalienne transitoire de 37%, ce qui signifie une meilleure qualité de vie sans traitement ou une thérapie substantive transitoire [10].

Chez notre patient, la surrénalectomie unilatérale de la glande la plus hyper-fixante a permis un contrôle clinico-biologique de la maladie avec une nette amélioration du profil glycémique et tensionnel. L'évaluation actuelle après 4 ans de la chirurgie ne retrouvant pas de signe d'hypercorticisme clinique. Enfin il faut garder à l´esprit l'éventualité d'une récidive nécessitant une totalisation chirurgicale, ce qui justifie un suivi régulier des patients avec une évaluation biologique et morphologique régulière.

## Conclusion

Le diagnostic d´hyperplasie macronodulaire bilatérale des surrénales est relativement aisé devant un tableau clinico-biologique d´hypercorticisme ACTH indépendant et un bilan morphologique bien conduit. Le traitement reste principalement chirurgical. Dans ce sens, les récentes études et publications optent pour une surrénalectomie unilatérale; efficace et moins pourvoyeuse de complications. Cependant, le risque de récidive et de survenue de complication cardiovasculaire justifie une surveillance au long cours de ces patients.
